# Army Legislation

**Published:** 1896-12

**Authors:** 


### A FEW THOUGHTS ON ARMY LEGISLATION.

We are now face to face again with this ever-important subject and, until the final battle shall have been won and the victory ours, there can be no cessation of activity. We have a committee fully alive to the importance of this measure, and well acquainted with work of this kind, willing to give time, money, effort, and all honorable means for success; determined that on their heads will fall the honors of leading to successful issue this long and faithfully fought warfare. With such a body to lead us there must be a hearty second from every organized body of veterinarians in the land and the personal assistance of every true devotee of the profession. No great achievements have ever been won without untiring energy, undaunted courage, and perseverance; and our efforts to secure this much-to-be-desired legislation can be well said to be no exception. Therefore, let everyone be up and doing, and let every Senator and Representative be sought out and labored with in the interest of this measure. Personal solicitation is the most successful plan of work, and before the new year as many should be seen as possible. There is one point that must not be overlooked in this battle, and that is the position of the army veterinarians. From some there have been the warmest and most earnest support and work, and they have only ceased when the last hours of Congress have been reached. Others who have given the measure indifferent support, and some from whom better things were expected, have been ostensibly in favor of the measure, but in reality against it. Some have persistently fought this legislation in every shape in which it has been presented, and they must now know that, with or without their support, this legislation must be engrafted upon our nation's statutes. The United States will only for a little longer remain the only great and enlightened country that has failed to grant rank to her veterinarians in her military service. The spirit of progress is abroad, and let they who run read the signs of the times.

